# CXCL1-LCN2 paracrine axis promotes progression of prostate cancer via the Src activation and epithelial-mesenchymal transition

**DOI:** 10.1186/s12964-019-0434-3

**Published:** 2019-09-10

**Authors:** Yongning Lu, Baijun Dong, Fan Xu, Yunze Xu, Jiahua Pan, Jiajia Song, Jin Zhang, Yiran Huang, Wei Xue

**Affiliations:** 1 Department of Urology, Renji Hospital, School of Medicine, Shanghai Jiao Tong University, No. 160 Pujian Road, Shanghai, 200127 China; 2 Reproductive Medicine Centre, Zhongshan Hospital, Fudan University, No.180 Fenglin Road, Shanghai, 200032 China

**Keywords:** Chemokine, Cytokine, Tumor cell migration, Tumor metastasis, Tumor microenvironment

## Abstract

**Background:**

Mechanisms driving the progression of castration-resistant prostate cancer are believed to relate substantially to the tumor microenvironment. However, the cross-talks between tumor epithelial cell, stromal cells, and immune cells are yet to be fully elucidated. The present study aims to determine the role of chemokine and neutrophil derived cytokine paracrine axis in mediating the interaction between tumor cells, stromal myofibroblasts, and neutrophils in the tumor microenvironment of prostate cancer.

**Methods:**

To identify myofibroblasts and neutrophil derived specific proteins affecting progression of prostate cancer, bioinformatics analyses were firstly performed in independent human prostate cancer gene expression data sets from the GEO data bank. Expression of stromal myofibroblasts secretory chemokine CXCL1 and neutrophil derived cytokine LCN2 was evaluated in prostate tissues via immunohistochemistry assay. We further investigated the effect of CXCL1 and LCN2 on prostate cancer using in vivo and in vitro models, and explored the underlying signal transduction pathways.

**Results:**

A CXCL1-LCN2 paracrine network was confirmed in prostate cancer tissue samples, which was correlated with the biochemical recurrence of prostate cancer. Of note, CXCL1-LCN2 axis activates Src signaling, triggers the epithelial-mesenchymal transition (EMT), consequently promotes the migration of prostate cancer cells, leading to enhanced tumor metastasis.

**Conclusions:**

Our findings may provide enhanced insight into the interactions of carcinoma-stromal cells and immune cells linked to prostate cancer progression, wherein CXCL1-LCN2 axis is a key contributor to prostate cancer cells migration. These data indicate tumor microenvironment and Src signaling pathway may be potential therapeutic targets of prostate cancer treatment.

## Background

Prostate cancer (PCa) is one of the most common malignant tumors, with the second-highest cancer-related mortality among men worldwide [1]. The appearance of castration-resistant prostate cancer (CRPC) or metastasis is often a sign of ultimate ultimately cancer mortality [2]. Development of CRPC or metastasis can be attributed to intrinsic cancer cell transformation or a combination of tumor and host interaction [3, 4]. Increasing evidence indicates that both tumor microenvironment and tumor-associated inflammation play critical roles in disease progression. Cytokines, growth factors, as well as matrix metalloproteinases secreted by stromal fibroblasts and myofibroblasts, are believed to support carcinoma cell survival even under low androgen conditions and promote both local tumor growth and distant metastasis [5, 6]. Moreover, chemokines (i.e., CXCL8/IL-8, CXCL12) overexpressed in prostate carcinoma activate proliferation-related pathways and stimulate angiogenesis, recruit immune cells infiltrating into cancerous foci, and trigger tumor-associated inflammation [7, 8].

CXCL1, a CXC family chemokine, is a newly discovered molecule to be overexpressed in PCa [9]. The PCa metastasis secretome study further suggests CXCL1 functions as a paracrine factor and may play a role in PCa bone metastasis by mediating cross-talk among multiple cell types within the tumor microenvironment [9, 10]. Recent study indicates that obese patients with PCa have increased CXCL1 expression. Moreover, CXCL1 overexpression is essential for the obesity-dependent tumor adipose stromal cells recruitment and ultimately promotes PCa progression [11]. Considering the primary biological function of this chemokine, it’s likely that overexpressed CXCL1 also recruits neutrophil to tumor microenvironment, which was proven in some other types of cancer such as breast cancer [12–14]. Tumor-associated inflammation and immune-cells like macrophages infiltration also contribute to PCa progression [15]. It has been found that neutrophils play a critical role in cancer biology and tumor development as well [16, 17]. A high neutrophil-to-lymphocyte ratio is a significant predictor of decreased PCa-specific survival and overall survival in patients that underwent radical prostatectomy [18]. Nevertheless, the role of tumor associated neutrophil on PCa progression is still unclear. As CXCL1 receptors CXCR1 and CXCR2 are expressed in both PCa cells and neutrophil, this chemokine may be a critical link in tumor microenvironment [19]. Here we questioned whether overexpressed CXCL1 in PCa as well as potentially recruited neutrophil might exert any influence on tumor cells by paracrine cytokine secretion. However, an integrated understanding of PCa progression in the context of input from cancer cells together with associated stromal cells and immune cells requires further investigation.

This study started with our interest in analyzing the potential link between CXCL1 and neutrophil-derived cytokines in the context of PCa. This effort led us to discover a network composed of PCa cells, stromal cells, and immune cells, which render a paracrine axis-centered chemokine CXCL1 and inflammatory cytokine LCN2 in conferring malignant phenotypes to PCa cells.

## Methods

### Bioinformation analysis

Eighteen independent microarray data sets of primary PCa (GSE7307, GSE6369, GSE37199, GSE36135, GSE35373, GSE33455, GSE3325, GSE32982, GSE32448, GSE26910, GSE22606, GSE21887, GSE17951, GSE17906, GSE17483, GSE17482, E-TABM-948, and E-MEXP-1243) were collected from Gene Expression Omnibus (GEO). Significance of correlation between CXCL1 (probe set 204470_at) and other genes was evaluated by Pearson’s correlation and Rank aggregation from all of the data sets. Protein products of genes that function extracellularly were chosen by filtering out genes that do not belong to the Gene Ontology Category Extracellular Space (GO: 0005615).

### Human prostate samples and immunohistochemistry (IHC) assay

Primary human prostate specimens were collected from 118 PCa patients from Renji Hospital. Tumor differentiation was defined according to the Gleason grading system. Tumor staging was defined according to the 7th edition of the American Joint Committee on Cancer TNM staging manual [20].

Tissue slides were stained with primary antibody against AMACR (ab219309), CXCL1 (ab86436), LCN2 (ab125075) from Abcam (Cambridge, UK), Keratin18 (#4548, Cell Signaling Technology, Massachusetts, USA) and CD177 (LS-B1953, LifeSpan BioSciences, Washington, USA). IHC results were evaluated independently by two pathologists who were blinded to the patients’ details. The staining intensity was scored on the following scale: negative (0), weak (1), moderate (2), or strong (3). The staining extent score was on a scale of 0–3, corresponding to the percentage of immunoreactive cells (< 5, 5–25%, 26–50%, 50–100%, respectively). An aggregate score ranging from 0 to 6 was calculated by adding the intensity score and staining extent score together, resulting in a negative (0–1), low (2), moderate (3–4), and high (5–6) expression for each specimen. PerkinElmer Opal™ Tyramide Signal Amplification system was employed for multiplex protein co-staining and images were captured by VectraPolaris™ Quantitative Slide Scanner (PerkinElmer, Massachusetts, USA).

### Cell culture

Human prostate adenocarcinoma cell lines LNCaP, PC-3, and DU145 were purchased from American Type Culture Collection. The normal human prostate epithelial cell line RWPE-1 and human myofibroblast stromal cell line WPMY-1 were purchased from Shanghai Institute for Life Science, Chinese Academy of Sciences. Recombinant human CXCL1 (275-GR) and LCN2 (1757-LC) from R&D SYSTEMS (Minnesota, USA) were used to stimulate cells. Signaling pathway inhibitors PP2 (S7008) and XAV939 (S1180) were purchased from Selleck (Texas, USA).

Conditioned medium (CM) and Transwell® co-culture systems.

One day before Transwell® co-culture experiments, tumor cells were plated in 6-well plates, WPMY-1 or freshly isolated normal human peripheral blood neutrophils were seeded into 6-well inserts (1.0 μm, Merk-Millipore, Damstadt, Germany). Inserts were plated into 6-well plates next day. Supernatant and cells were collected after 72 h.

For the CM co-culture system, WPMY-1 cells were plated in 10-cm-diameter dishes in complete growth medium which was replaced with conditioned or control medium after 24 h. In tumor cells activated groups, WPMY-1 was treated with CM derived from various PCa cell lines (50% freshly collected serum-free supernatant from tumor cell culture + 50% DMEM medium, 1% FBS); in control group, WPMY-1 was cultured in fresh medium supplemented with 1% FBS. After 24-h treatment, WPMY-1 was rinsed with PBS and cultured in 5 mL fresh DMEM medium for another 24 h. CM derived from various groups of WPMY-1 were collected and stored at − 80 °C. In treated groups, tumor cells were cultured in CM obtained either from stimulated or control WPMY-1 for 72 h (CM: tumor cell culture medium = 1:1).

### Cell proliferation assays

Tumor cells were seeded into 96-well plates overnight and treated with CM or various concentrations of cytokines for 72 h. Cell proliferation was assessed by sulforhodamine B (SRB) assay as previously described in literature [21].

### Transwell migration assays

Tumor cells in vitro migration assays were carried out using the Transwell® system (24-well inserts, 8.0 μm; Corning, New York, USA). After specific treatment, tumor cells were detached and re-suspended in serum-free medium. Cells were cultured on the top chambers for 18 h and growth medium was added to the bottom wells. In some cases, specific antibodies or inhibitors were added into both chambers. Quantification was performed by counting crystal violet stained migrated cells in five 200× high power fields (HPFs) of every top chamber.

### Isolation of neutrophils from human peripheral blood

Blood samples was collected in 5-ml EDTA-coated vacutainers from healthy male donors in our group and neutrophils were enriched using Percoll method as literature described [22]. This type of immune cells can be recognized by FITC-conjugated mouse anti-human CD15 antibody (Biolegend, 301,903) and the purity of cells was identified using flow cytometry.

### Bio-plex array

The levels of cytokines in cell culture supernatant was measured with Bio-Plex human cytokine panels (M500KCAF0Y, 171-W4001 M, YF0-00000AY, Bio-Plex Human Cytokine Group II 10-plex, Bio-rad, California, USA) and suspension array system according to the manufacturer’s manual.

### Raybiotech cytokine array

The relative expression levels of 507 cytokines, chemokines, and growth factors in the supernatant of co-culture systems were simultaneously detected using RayBio® L-Series Human Antibody Array 507 (Cat# AAH-BLM-1-4, Raybiotech, Georgia, USA) according to the manufacturer’s protocol.

### Elisa

The collected cell culture supernatant was measured for CXCL1 (R&D SYSTEMS, DGR00), MMP-LCN2 complex (R&D SYSTEMS, DM9L20), and LCN2 (R&D SYSTEMS, DLCN20) by ELISA following the manufacturer’s instructions.

### Immunoblotting

For western blot analysis, cells were lysed in RIPA buffer (Beyotime Institute of Biotechnology, Shanghai, China) supplemented with proteinase inhibitor cocktail (Roche, Basel, Switzerland). Protein expression was evaluated as previously described in literature [23].

### Quantitative Realtime-PCR

Total RNA was isolated from WPMY-1 cells using Total DNA/RNA/protein isolation kit (Omega Bio-tek, Georgia, USA). Quantitative real-time-PCR amplification was performed by using the ABI 7500FAST system according to manufacturer’s instructions. The expression of reference genes (beta-actin and GAPDH) was not affected by treatment and correlated with the amount of RNA used for reverse transcription. Data are presented as relative expression (RE): RE = 2ΔCt, ΔCt = Ct (target gene)-Ct (reference genes).

### Animals and in vivo metastatic study

Male nude mice (3–4 weeks old) were purchased and housed 1 week before experiment under pathogen-free conditions. All animal experiments were performed according to the institutional ethical guidelines of animal care. Mice were divided into three groups of 10 animals each. Tumor cells were constantly stimulated with CXCL1 or LCN2 for 5 days. To maintain the stimulation, culture medium supplemented with CXCL1 or LCN2 was replaced freshly every 24 h. After daily cytokine treatment, DU145 (control, CXCL1-treated, and LCN2-treated) suspension (1 × 10^6^/100 μl each mouse) was injected via the tail vein. Mice were killed 12 weeks after inoculation. Lung tissues were resected and fixed in 4% para-formaldehyde overnight at 4 °C before metastasis evaluation.

### Statistics

Measurement data are presented as mean ± standard deviation (SD) and statistical significance between groups was determined by ANOVA, Student’s *t*-test, or Mann–Whitney test (two-sided). Differences of cross-tables were determined by Fisher’s exact test. Correlations between factors were analyzed by Pearson’s test. Kaplan–Meier and log-rank test analyses were used to evaluate the association of CXCL1-LCN2 expression and biochemical recurrence-free survival. The univariate and multivariate Cox proportional hazards regression models were used to associate CXCL1 and LCN2 with clinicopathological data and biochemical recurrence. Differences were considered statistically significant at *p < 0.05, **p < 0.01 and ***p < 0.001.

### Study approval

Animal studies were approved in advance by the Animal Ethics and Experimentation Committee. Studies using patient samples were approved by the Renji Hospital Human Ethics Research Committee, and all healthy donors consented to the use of their peripheral blood for neutrophil isolation.

## Results

### Neutrophil-derived LCN2 is a highly related factor co-expressed with CXCL1 in prostate cancer

To identify molecules presumably involved in mediating the impact of CXCL1 and neutrophils, we first analyzed the published human PCa gene expression data sets, with a focus on neutrophil-derived genes encoding secreted proteins. Specifically, a list of CXCL1-associated candidates was stratified from 18 independent human PCa gene expression data sets from the GEO data bank (Additional file 1: Table S1), which highlighted IL8/CXCL8 and LCN2 as neutrophil-derived cytokines that were closely associated with CXCL1. LCN2 is mainly synthesized and secreted from neutrophil and plays a role in the innate immune response to bacterial infection by sequestrating iron from pathogens. Unlike IL8/CXCL8 which has been extensively investigated [8, 19, 24], only very few studies suggested LCN2 also involved in PCa progression [25]. The role of CXCL1 and LCN2 in PCa remains largely unexplored. We hence chose to focus on these two factors for further investigation.

### CXCL1-LCN2 co-expression pattern serves as an independent prognostic factor of biochemical recurrence after radical prostatectomy

**Fig. 1:**
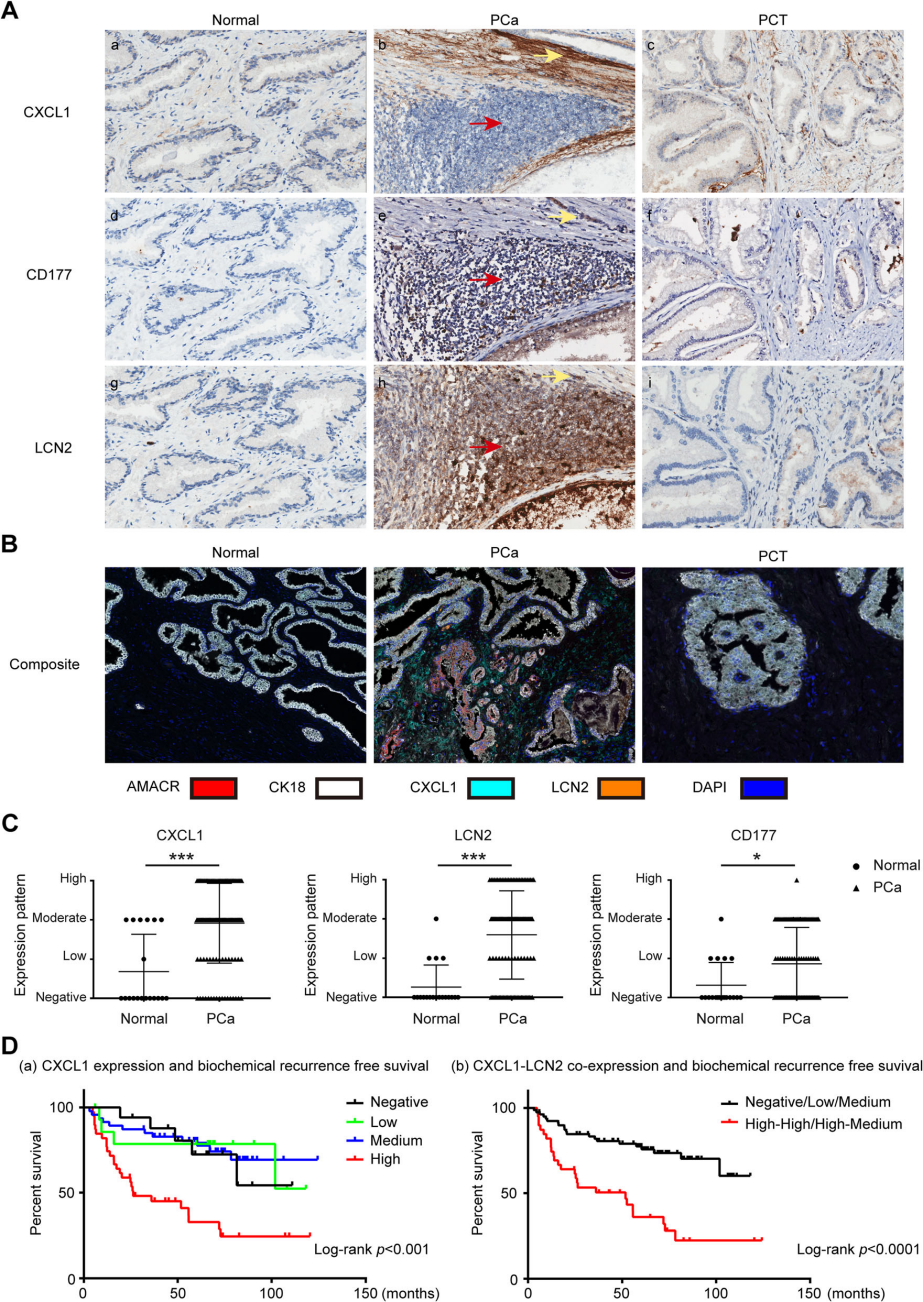
Expression of CXCL1, CD177 and LCN2 in prostate tissues and correlation with biochemical recurrence free survival in prostate cancer cases. **a** Representative images (magnification 200X) of CXCL1 (a~c), CD177 (d~f) and LCN2 (g~i) expression in normal prostate tissues, cancerous lesion and para-carcinoma tissue (PCT). Red arrows: tumor foci; yellow arrows: prostate stroma. **b** Representative images (magnification 200X) of AMACR (red, expressed in carcinoma epithelium), CK18 (white, expressed in prostate glandular epithelium), CXCL1 (cyan) and LCN2 (orange) co-staining in normal prostate tissues, carcinoma tissue and PCT observed by PerkinElmer Tyramide Signal Amplification system. **c** Semi-quantitative data of CXCL1, LCN2 and CD177 expression in normal prostate (*n* = 19) and prostate cancer (*n* = 118) are shown in scatter plots. *p* value was determined by Mann–Whitney test, asterisks indicate *p < 0.05, ***p < 0.001. **d** Kaplan-Meier and log-rank test were used to evaluate CXCL1 expression alone (a) or CXCL1-LCN2 co-expression (b) and biochemical recurrence free survival. High expression level of CXCL1 (CXCL1 High vs. Negative expression *p* < 0.001) and high co-expression of CXCL1-LCN2 (High-High/High-Medium vs. Negative/Low/Medium expression *p* < 0.0001) predicted early biochemical recurrence after radical prostatectomy

We next confirmed the expression patterns of CXCL1 and LCN2 in PCa using IHC assay. CXCL1, LCN2, and CD177—a specific antigen of neutrophil—were respectively stained in prostate samples from 118 cases with PCa and 19 cases of benign control. AMACR was labeled in malignant epithelium and Keratin18 (CK18) was used to indicate glandular epithelium in prostate. Clinical and pathological characteristics of PCa cases are presented in Additional file 1: Table S2. Notably, CXCL1 expression was much higher in tumor foci than para-carcinoma tissue (PCT) in the same PCa samples as well as unrelated normal prostate tissues (Fig. 1a and c, *p* < 0.001). Further, with a detailed look into CXCL1 localization within tumor foci, our results revealed that CXCL1 was predominately expressed in stromal cells (yellow arrows) instead of glandular epithelial tumor cells (red arrows, Fig. 1a; Cyan, Fig. 1b), suggesting that it mainly originates from stromal tumor-associated fibroblasts.

Likewise, CD177 staining, which indicates neutrophil infiltration within tissue, was predominately observed in tumor foci but was barely detectable in PCT or normal prostate tissue (Fig. 1a and c, *p* < 0.05). LCN2 levels showed a similar pattern as CD177 staining, significantly higher in tumor foci than PCT and normal tissue (Fig. 1a and c, *p* < 0.001), although it was expressed not only in prostate stroma but also in epithelial cells in tumor foci (Orange, Fig. 1b). Furthermore, using Opal™ multiplex IHC technique, the close connection of prostate carcinoma epithelium (indicated by AMACR), tumor related fibroblasts (labeled by CXCL1) and tumor related neutrophil (stained with LCN2) was evidenced in Fig. 1b. Importantly, the patterns of expression and distribution of CXCL1, LCN2 and CD177 were closely related to each other (Additional file 1: Table S3), supporting their functional relevance in PCa.

Further analysis indicated that the high expression level of CXCL1 alone predicted early biochemical recurrence after radical prostatectomy (high vs. negative CXCL1 expression, *p* < 0.0001) (Fig. 1d-a, Additional file 1: Table S4). Univariate and multivariate analysis with Cox regression showed CXCL1 to be an independent prognostic factor of biochemical recurrence after radical prostatectomy (Additional file 1: Table S5). Remarkably, high CXCL1-LCN2 co-expression pattern provides an optimized way to distinguish those cases in whom early biochemical recurrence is more likely to occur (Fig. 1d-b).

### Reciprocal interactions between PCa cells and myofibroblasts facilitate tumor proliferation and migration

Based on aforementioned clinical data, it is worthwhile to investigate the cross-talk between tumor cells and adjacent stromal cells, in particular fibroblasts. Hence, we used WPMY-1, a myofibroblast cell line from normal prostate cells as a representative of host stromal cells. CM from DU145 cells (CM-DU145) could stimulate proliferation of WPMY-1 (Additional file 1: Figure S1A). In line with this observation, the mRNA level of FAP-α in WPMY-1 cells was dramatically increased when co-cultured with DU145 cells in a Transwell® co-culture system, demonstrating the characteristics of tumor-associated fibroblasts (Additional file 1: Figure S1B).

**Fig. 2:**
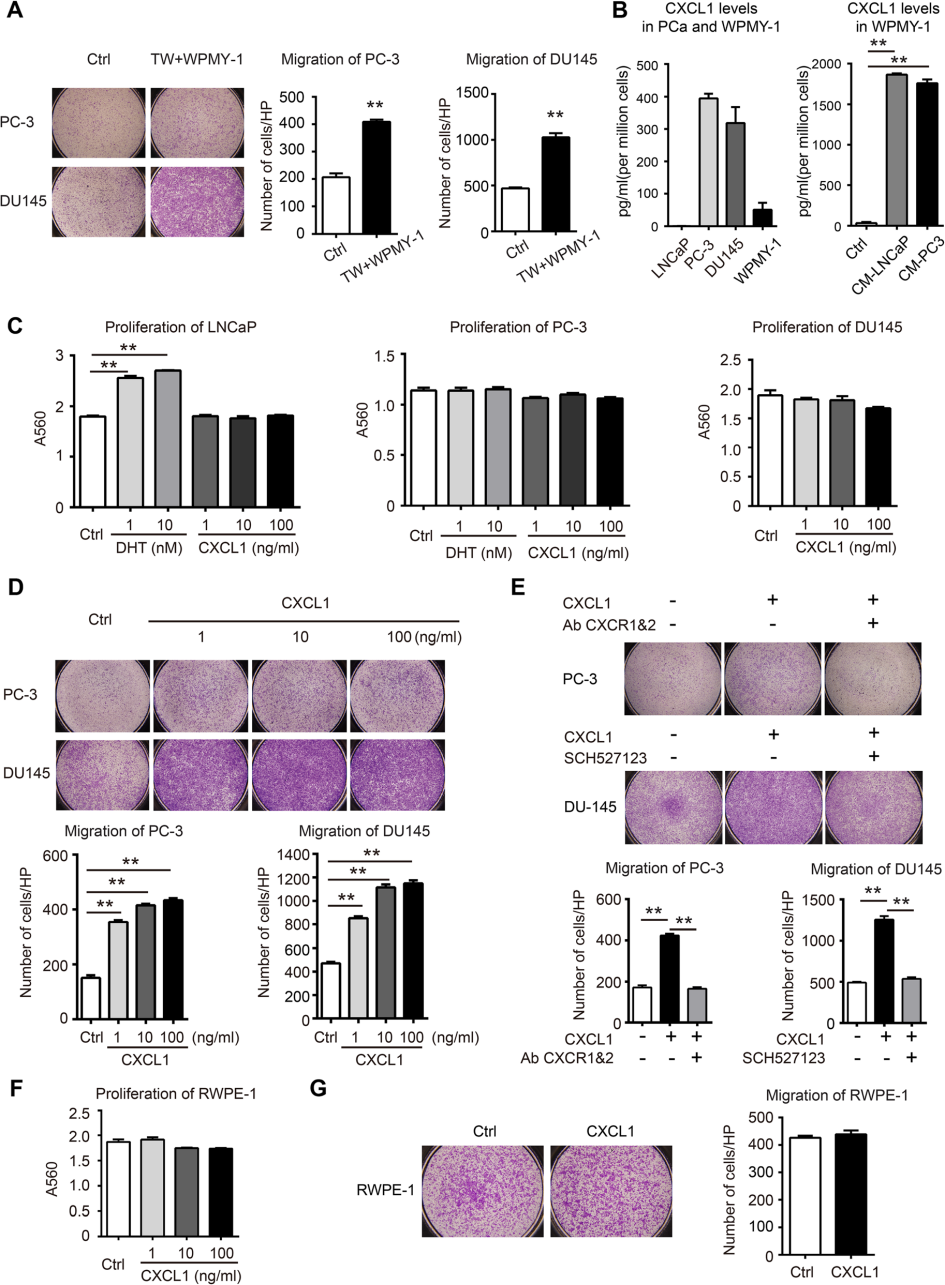
CXCL1 contributes to myofibroblast promoted migration in prostate cancer cells. **a** Representative images (magnification 40X) of cells migration in prostate cancer cells control and those co-cultured with WPMY-1 in transwell (TW) system. **b** CXCL1 secretion in PCa, naïve WPMY-1 and PCa CM treated WPMY-1 was determined using ELISA. This cytokine concentration was normalized with numbers of cells. **c** Influence of CXCL1 on prostate cancer cells proliferation was determined using SRB. DHT was used as control to treat androgen-sensitive cell line LNCaP and androgen-insensitive PC-3. **d** Representative cells migration images (magnification 40X) of PC-3 and DU145 treated with different concentrations of CXCL1. **e** Representative images (magnification 40X) of cells migration in prostate cancer cells control and those treated with CXCL1, with or without supplement of CXCR1/2 blockage using specific antibodies or inhibitor. **f** Influence of CXCL1 on benign prostate epithelial cell RWPE-1 proliferation was determined using SRB. **g** Representative cells migration images (magnification 40X) of RWPE-1 treated with CXCL1. Data are represented as mean ± SD from a representative triplicate experiment. Histograms shows quantitative results and *p* value was determined by ANOVA or t test, asterisks indicate *p < 0.05, **p < 0.01

Next, we used WPMY-1–based co-culture systems to study the interaction between tumor-associated fibroblasts and tumor cells. The impact of tumor-stimulated WPMY-1 on proliferation of cancer cells was evaluated. Three PCa cell lines (i.e., LNCaP, PC3, and DU145) were stimulated by WPMY-1 culture medium, which was pre-treated with CM of corresponding tumor cells. Indeed, proliferation of all tumor cell lines was unanimously promoted by WPMY-1 culture supernatant (Additional file 1: Figure S1C). Interestingly, this effect was even more remarkable than the stimulation of culture medium from naïve WPMY-1. We also measured the migration ability of cancer cells, which was substantially enhanced after being co-cultured with WPMY-1 for 3 days, and particularly significant in PC-3 and DU145 (Fig. 2a).

To gain insights into the molecular basis between tumor-stromal cross-talk observed in this study, we performed a bio-plex array to profile secreted cytokines in co-culture systems. Indeed, a set of cytokines and chemokines profoundly increased in WPMY-1 pre-treated with CM of PC-3 compared to naïve WPMY-1 (Additional file 1: Figure S1D). Likewise, a similar pattern was observed in the supernatant of WPMY-1 cells co-cultured with LNCaP cells when compared with either naïve WPMY-1 or LNCaP cells alone (Additional file 1: Figure S1E). Among these increased cytokines, CXCL-1 was profoundly increased in both co-culture systems, which was subsequently confirmed using ELISA (Fig. 2b).

### CXCL1 promotes migration of PCa cells

We next examined whether CXCL-1 was functionally essential in mediating tumor and stromal cross-talk. To this end, LNCaP, PC3, and DU145 PCa cell lines were directly stimulated by recombinant human CXCL1 for 72 h. While cell proliferation was barely affected (Fig. 2c), we observed significant enhancement in the migration ability of the PC-3 and DU145 cells resulting from CXCL1 treatment in a dose-dependent manner (Fig. 2d). For further confirmation, CXCL1-induced promotion of tumor cell migration was abolished by neutralizing antibodies against CXCR1/2—two classical chemokine receptors for CXCL1 or selective CXCR1/2 antagonist SCH527123 (Fig. 2e). Interestingly, neither proliferation stimulation nor migration enhancement was found in CXCL1-treated normal prostate epithelial cell line RWPE-1 (Fig. 2f–g). These results together suggest the essential role of CXCL1 in mediating tumor-stromal cross-talk by specifically promoting the migration of PCa cells.

### LCN2 secretion by neutrophils promote PCa cell migration

**Fig. 3:**
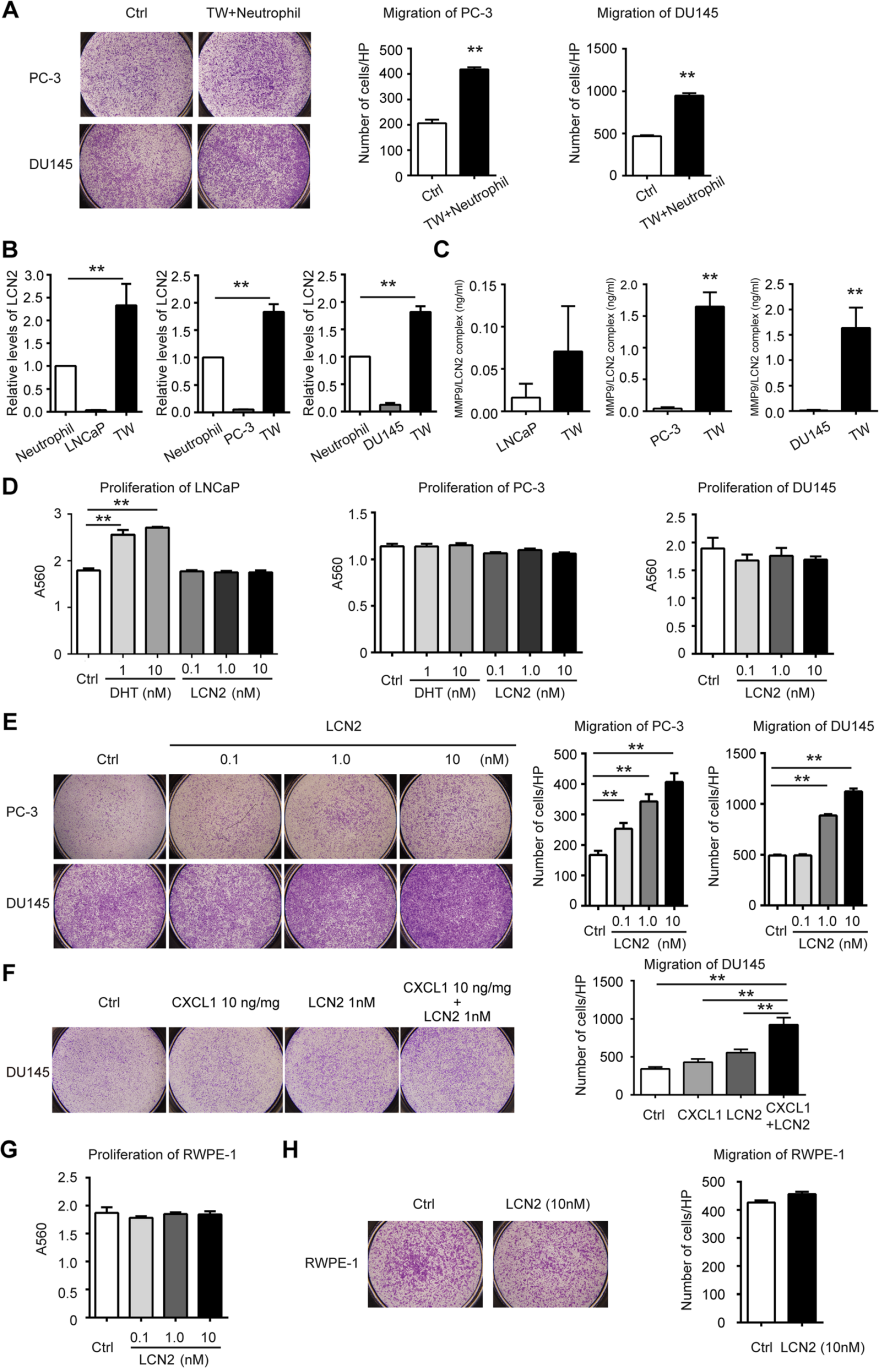
Prostate cancer cell-neutrophil interaction in transwell systems and the influence of LCN2 on tumor cells. **a** Representative cells migration images (magnification 40X) of PC-3 and DU145 co-cultured with neutrophil. **b** LCN2 secretion in neutrophil, tumor cell lines and co-cultured system were determined using ELISA. The LCN2 level of neutrophil control was normalized as 1. **c** MMP9/LCN2 secretion in tumor cell lines and respective co-cultured system with neutrophil were measured using ELISA. **d** Influence of LCN2 on prostate cancer cells proliferation was determined using SRB. DHT was used as control to treat androgen-sensitive cell line LNCaP and androgen-insensitive PC-3. **e** Representative cells migration images (magnification 40X) of PC-3 and DU145 treated with different doses of LCN2. **f** DU145 was treated with low concentration of CXCL1/LCN2 separately or in combination for 96 h. **g** Influence of LCN2 on benign prostate epithelial cell RWPE-1 proliferation was determined using SRB. **h** Cells migration of RWPE-1 treated with LCN2. Representative images (magnification 40X) and quantitative data were shown. Data are represented as mean ± SD of triplicates from a representative experiment of *n* = 3. Histograms shows quantitative results and *p* value was determined by ANOVA or t test, asterisks indicate *p < 0.05, **p < 0.01

Although evidence of infiltration of neutrophils was found in tumor foci (Fig. 1a d–f), it is still unclear whether this type of immune cell might further strengthen progression of PCa. To test this hypothesis, normal human neutrophils (hNEP) from healthy male donors were isolated, purified, and co-cultured with prostate tumor cells in the Transwell® system for 72 h. It was found that migration ability of PC-3 and DU145 was significantly promoted when co-cultured with hNEP (Fig. 3a).

Further, to confirm our IHC data from PCa tissue, the presence of LCN2 in the supernatant of hNEP-PCa cells co-culture was further measured. LCN2 was detected in the supernatant of both hNEP and tumor cells, in which hNEP secreted over 10 times amount of LCN2 than the same number of tumor cells (Fig. 3b), suggesting hNEP as the major source of LCN2 in the tumor microenvironment. Importantly, nearly double amount of LCN2 was secreted in the co-culture compared with that from hNEP alone, which was consistently observed in all co-culture groups. Moreover, we observed increased levels of MMP9/LCN2 complex in all co-culture systems, especially with highly invasive cell lines—PC-3 and DU145 present (Fig. 3c). Overall, we found that CXCL1-associated neutrophil recruitment may lead to local LCN2 enrichment and MMP9/LCN2 complex increase in the PCa microenvironment.

We next investigated the biological impact of LCN2. PCa cells were stimulated by recombinant human LCN2 for 72 h. Similar to CXCL1, LCN2 treatment did not show a notable impact on cancer cell proliferation (Fig. 3d). Remarkably, it was also found that migration activity of PC-3 and DU145 cells was dramatically enhanced by LCN2 in a dose-dependent manner (Fig. 3e). Notably, in DU145 treated with a combination of CXCL1 and LCN2, the additional enhancement of cell migration was observed compared with each single stimulation (Fig. 3f). However, this effect also seems exclusively observed in tumor cells instead of RWPE-1 (Fig. 3g and h).

### CXCL1 and LCN2 facilitate PCa cell metastasis in vivo

**Fig. 4:**
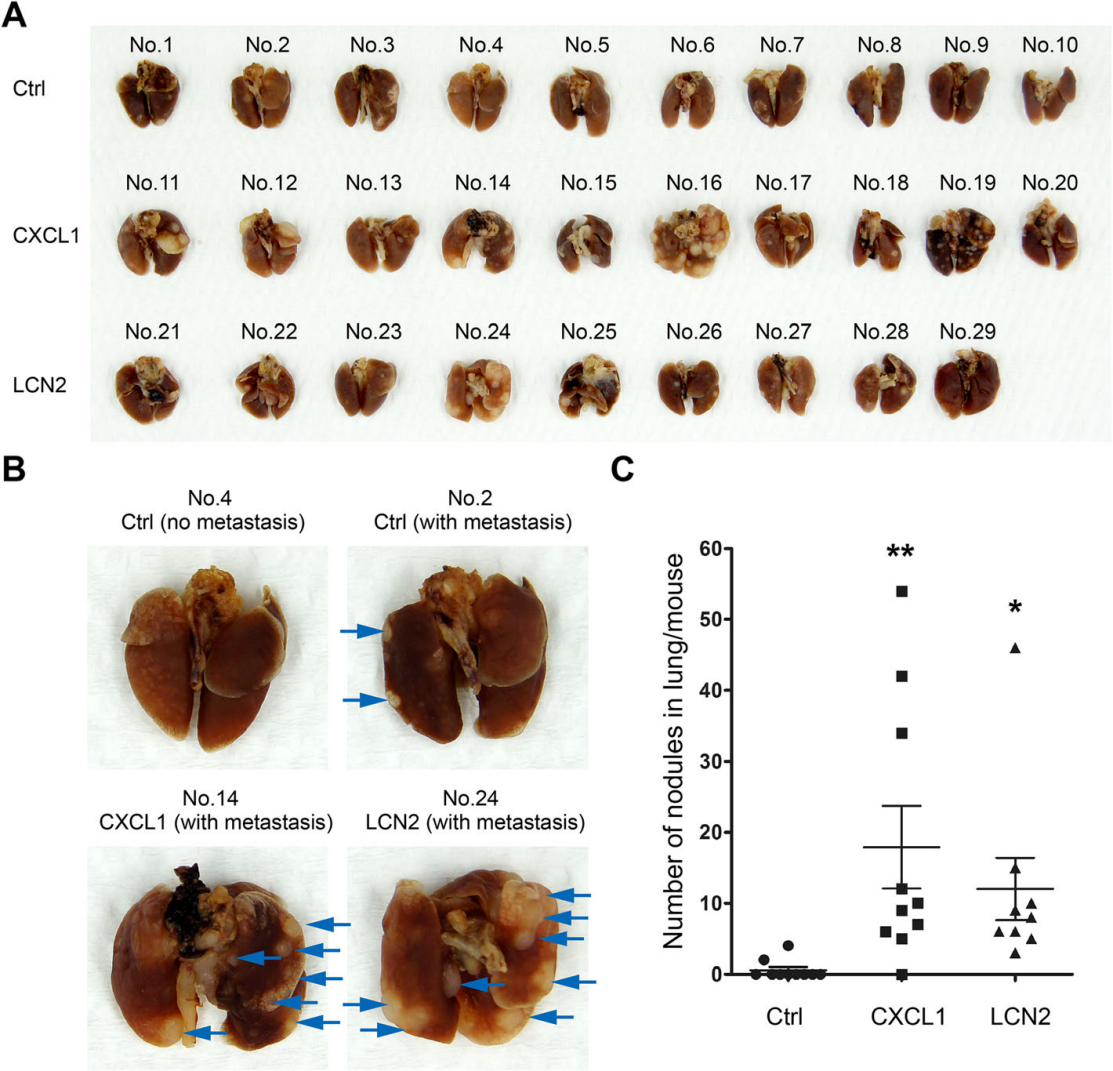
CXCL1&LCN2 facilitate prostate cancer cells metastasis in mice model. **a** Lung tissues taken from 29 mice inoculated with control, CXCL1 or LCN2 treated DU145cells via tail vein injection. **b** Representative lung samples of three groups demonstrating tumor metastasis nodules indicated with blue arrows. **c** Comparison of the numbers of metastatic nodules in each mouse between CXCL1 or LCN2 treated group with control. *p* value was determined by ANOVA test

Cell migration is essential for cancer metastasis. With a mice metastasis model, we discovered that incidence of lung metastasis in CXCL1 or LCN2-treated group was significantly higher than in untreated group (20% in untreated group, 90% in CXCL1-treated group, and 100% in LCN2-treated group; Fig. 4a). Likewise, the numbers of metastatic nodules in individual lung tissue samples were profoundly increased in mice inoculated with CXCL1- or LCN2-treated DU145 (Fig. 4b and c).

### CXCL1-LCN2 promotes PCa cells migration via Src family kinases activation and epithelial-mesenchymal transition (EMT)

**Fig. 5:**
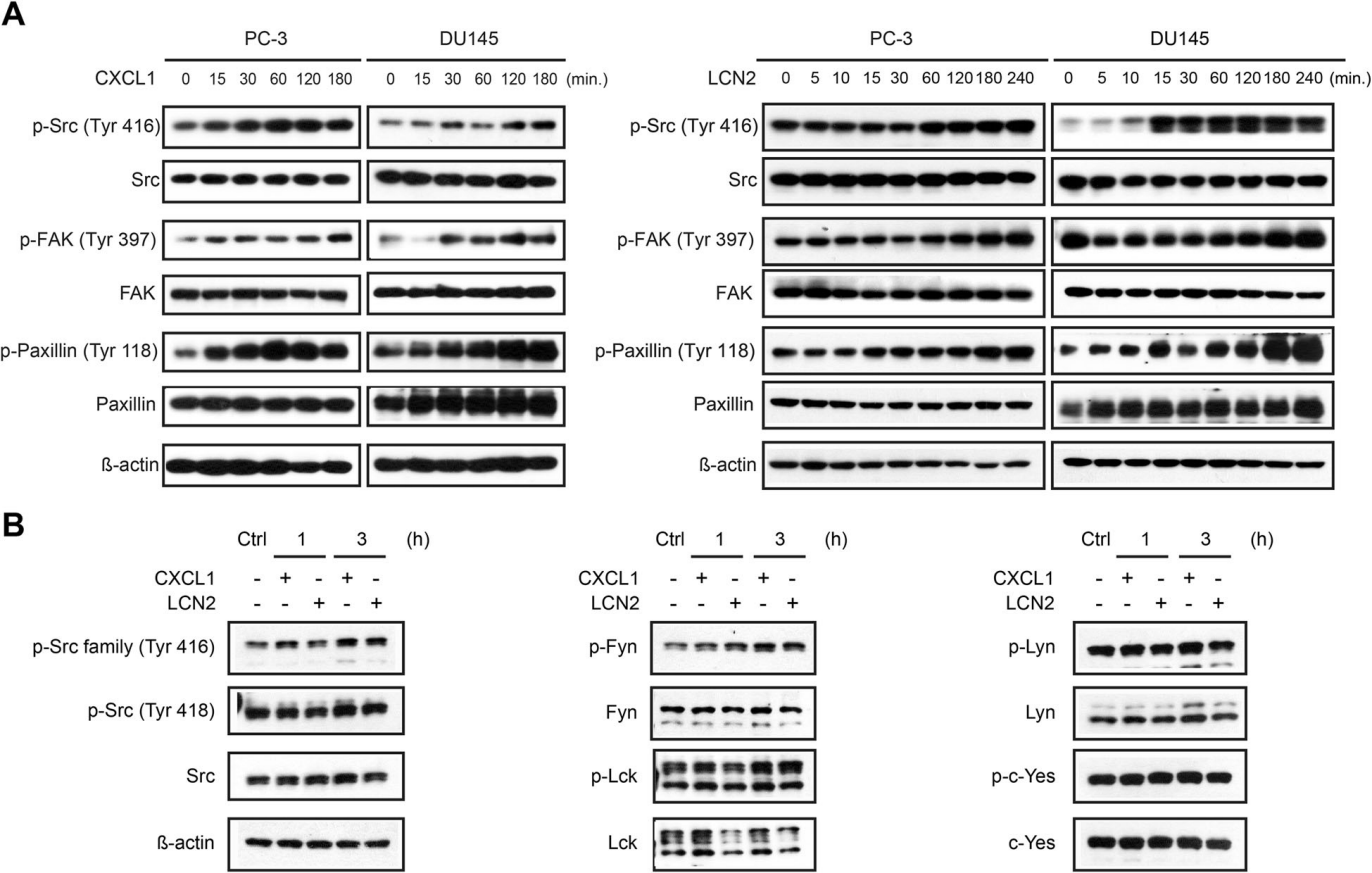
Src family kinases activation in CXCL1 or LCN2 treated prostate cancer cells. **a** Activation of Src-FAK-Paxillin pathway was indicated by phosphorylation of these kinases in CXCL1 or LCN2 treated PC-3 and DU145 in time-dependent manner. **b** Phosphorylation of Src family members (Src, Fyn, Lck, Lyn, c-Yes) in CXCL1&LCN2 treated prostate cancer cells was measured using western-blotting. Representative blots from at least twice independent experiments are shown

It has been well established that Src family kinases (SFKs) signaling pathway plays a critical role in driving migration and metastasis of a variety of cancers including PCa [26, 27]. We hence explored possible connection between the CXCL1-LCN2 axis and SFKs signaling. It was observed that both CXCL1 and LCN2 treatment of PCa cells increased phosphorylation of SFKs, as detected by a tyrosine 416 phosphorylation-specific, pan-SFK antibody, as well as the downstream modulators FAK and Paxillin (Fig. 5a). Consistently, the selective CXCR1/2 antagonist—SCH527123—was able to abolish CXCL1-induced Src/FAK-Paxillin activation to a certain extent (Additional file 1: Figure S2).

**Fig. 6:**
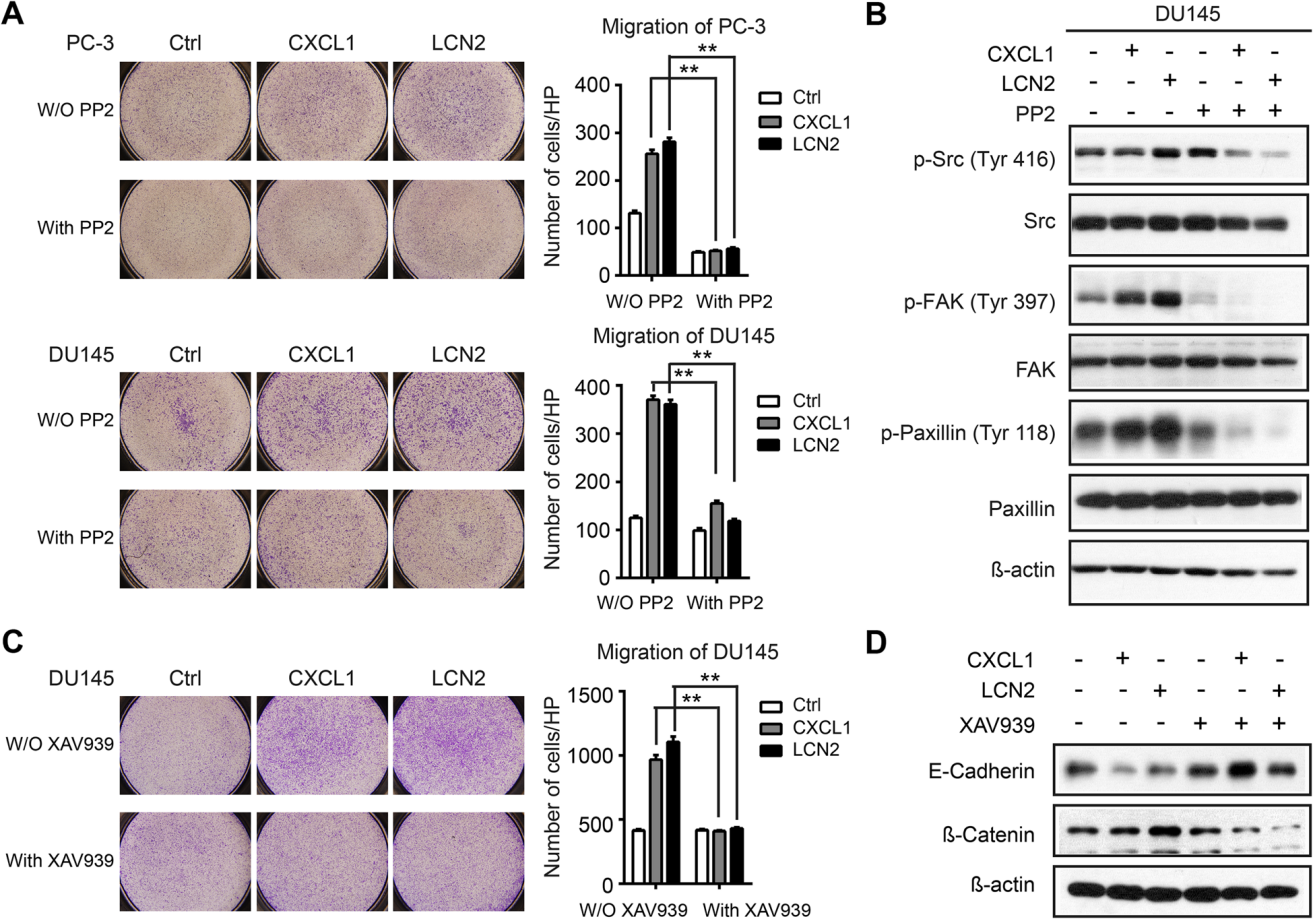
Src family kinases activation in CXCL1 or LCN2 treated prostate cancer cells mediates cancer cells migration. **a** Representative images (magnification 40X) of prostate cancer cells migration after treatment of CXCL1 or LCN2 with or without SFKs inhibitor PP2. Histograms shows quantitative results from three times experiments and *p* value was determined by Student’s t test. **b** Phosphorylation of Src-FAK-Paxillin in DU145 after treatment of CXCL1 or LCN2 with or without supplement of PP2 was detected using western blotting. **c** Representative cells migration images (magnification 40X) of DU145 stimulated with CXCL1 or LCN2 in addition of β-Catenin inhibitor XAV939. **d** Expression of EMT markers in DU145 after CXCL1/LCN2 treatment was determined using western blotting

To further specify which SFKs were specifically involved, we examined phosphorylation in major tyrosine sites of several SFKs members (i.e., Src, Fyn, Lck, Lyn, and c-Yes) in CXCL1- and LCN2-treated cells. We found that only phosphorylation of Fyn responded to CXCL1 or LCN2 treatment (Fig. 5b). Moreover, PP2, a specific SFKs inhibitor mainly targeting Lck/Fyn and displaying anticancer activity [28, 29], abrogated the migration of CXCL1/LCN2 stimulated tumor cells (Fig. 6a and b). These results suggested that Fyn signaling is essential to mediate the impact of CXCL1 and LCN2 in promoting the migration of PCa cells.

Moreover, consistent with the enhancement of the migration in PCa cells stimulated with CXCL1 or LCN2 for 96 h, the down-regulation of E-Cadherin and up-regulation of ß-catenin was noted, indicating that EMT may take place after long-term stimulation. Interestingly, XAV939, a specific inhibitor of ß-catenin conducting transcription [30], restored E-Cadherin expression and abolished migration enhancement in PCa cells (Fig. 6c and d).

## Discussion

The major impediments to treating advanced PCa are the development of widespread metastasis and resistance to androgen deprivation therapy. Recently, it has been increasingly recognized that a comprehensive understanding of the role of the tumor microenvironment regarding tumor progression is essential to improve clinical outcomes in PCa treatment [5, 29]. This study sheds light on tumor-associated inflammation and tumor progression by investigating the interaction between PCa cells and surrounding cells. Our results point to a new paracrine activation loop between PCa cells, stromal myofibroblasts, and neutrophils involving CXCL1 and LCN2, which are shown to confer a more invasive phenotype to cancer cells by activating Src family kinases and EMT. These results support a working model that CXCL1 release occurs when myofibroblasts are associated with PCa cells, which further drives the enrichment of neutrophil-derived LCN2 and together enhances cancer cells migration.

Previous studies have revealed that primary human prostatic stromal cells do not affect the growth of prostate epithelial cell lines but stimulate its motility [31]. In the present study, CM from naïve WPMY-1 only slightly promoted cancer cells proliferation whereas this effect was dramatically magnified when stromal cells were pretreated with culture supernatant from tumor cells. Of note, it appears that the stimulating effect of co-culture on LNCaP is not as prominent as observed in the PC3 and DU145 cells, which are androgen insensitive and more aggressive cell lines. These results indicate that prostate tumor cells, especially those with a high-malignant phenotype, may be more likely to take advantage of the tumor microenvironment for survival and metastasis by reciprocally interacting with stromal cells.

In order to collect and investigate cytokines in culture medium as well as different types of cells separately after co-culture experiment, we used Transwell® system instead of 3D co-culture. Interestingly, current findings suggest that indirect contact between PCa cells and myofibroblasts induces the release of a storm of cytokines and chemokines including CXCL1, IL-6, and IL-8/CXCL8. Recent reports show that Kallikrein-related peptidase-4 from PCa cell culture supernatant stimulates IL-6 production from WPMY-1, which is an upstream regulator of CXCL1 expression [32, 33]. In this study, increased IL-6 may further boost CXCL1 secretion in cancer cells–WPMY-1 co-culture systems. Unlike the chemotactic effects of CXCL1 on PCa cells reported by Kuo et al. [34], present study demonstrates that CXCL1 stimulation predisposes tumor cells to a more aggressive behavior by binding to specific receptors—CXCR1/2. More importantly, for the first time, our data show that high CXCL1 expression in the stroma of prostatic cancerous foci is an independent prognostic factor of early biochemical recurrence after radical prostatectomy. These data suggest that CXCL1 may be a promising biomarker to distinguish high-risk PCa.

As a primary target cell type of CXCL1, neutrophil plays an obscure role in the tumor microenvironment and have thus become a focus of research only in the last few years. In spite of its well-recognized shorter lifespan than monocytes/macrophages or lymphocytes, the role of neutrophils in tumor-associated inflammation is still worthy of thorough investigation, in particular considering its abundance among white blood cells [35]. LCN2 is a proinflammatory cytokine that can be secreted by activated neutrophils, and it was recently found to be overexpressed in various types of solid tumors [36, 37]. It has been reported that LCN2 can form a complex with matrix metalloproteinase-9 (MMP-9), thereby preventing MMP-9 degradation and enhancing its activity in vitro [38], which may also enhance tumor invasion. Newly published studies show that overexpression of LCN2 in PCa cell lines promote cell motility and invasiveness [25, 39]. However, our in vitro data confirm that instead of cancer cells themselves, neutrophils, especially those co-cultured with cancer cells, are the major source of LCN2 and may contribute to the abundant LCN2 in tumor foci. This was consistent with the identified correlation of CXCL1-CD177-LCN2 in cancerous foci of the prostate in our clinical samples, shown by Opal™ multiplex IHC technique.

Furthermore, our work demonstrates that both increased CXCL1 and LCN2 in the tumor microenvironment confers a more aggressive phenotype upon cancer cells via the activation of Src family kinases. Hyperactivation of Src kinase is displayed in previous studies and is responsible for inappropriate activation of androgen receptors by nonsteroids in PCa [40, 41]. On the other hand, our data indicates that long-term exposure of PCa cells on CXCL1 and LCN2 also triggers EMT, which is a well-known speculated mechanism of tumor metastasis [42, 43]. As shown in our study, it likely explains the enhancement of PCa metastasis in mice model. Of note, CXCL1 and LCN2 synergistically enhance tumor malignancy, indicated by the profound promotion of DU145 migration that occurs from the stimulation with both cytokines. Together with our findings, this implies that CXCL1-LCN2 paracrine axis may trigger a cascade amplification event that promotes PCa metastasis. Moreover, the present study indicates that CXCR1/2 antagonist and Src family inhibitors or ß-catenin inhibitors may be promising pharmacological approaches in the treatment of advanced prostate carcinoma. Although a previous phase II clinical trial using Src kinase inhibitor, saracatinib, to treat PCa was terminated, which is likely due to the low rate of randomized patients [44], it is still worthy to further explore the application of these kinds of inhibitor for PCa treatment [45].

Our study still has some limitations. First, it was seen that CXCL1- or LCN2-treated PCa cells showed much higher invasiveness in a nude mice tail vein injection model. Nevertheless, it would be more convincing to investigate in vivo tumorigenesis promoting effects of stromal cells or neutrophils using reliable animal models in which CXCL1 or LCN2 from the above cells can be functionally silent or abrogated. Second, it is still unclear how LCN2 activates Src signaling in PCa cells, considering LCN2 neutralizing antibody was not available. These issues require further detailed investigation in future studies.

## Conclusions

**Fig. 7:**
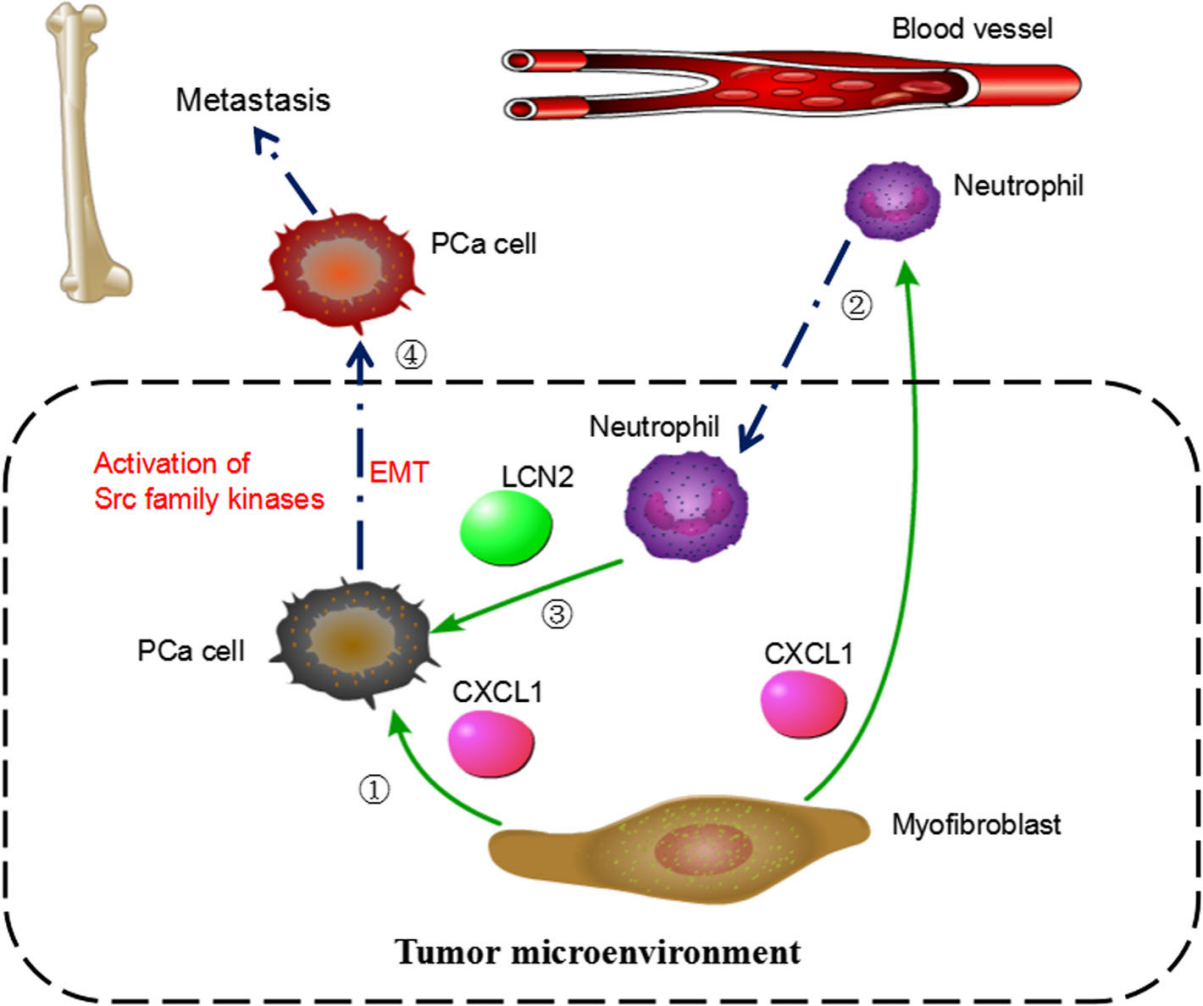
Schematic diagram of CXCL1-LCN2 paracrine axis in prostate cancer microenvironment. CXCL1 secreted from myofibroblast directly enhances prostate cancer cell migration (①); CXCL1 recruits neutrophils to prostate cancerous lesion (②); LCN secreted from neutrophil further promotes tumor cells migration (③); Activation of Src family kinases contributes to highly aggressive phenotype of prostate cancer cells stimulated by CXCL1 and LCN2 (④)

Taken together, our results provide mechanistic insights into the interaction between PCa cells, stromal cells, and innate immune cells in tumor microenvironment and highlight the role of CXCL1 and LCN2 in a tumor-stroma paracrine axis in promoting cancer-cell aggressiveness (Fig. 7). This study raises the possibility of clinically targeting this loop and related Src pathway to treat advanced PCa. Based the current investigation, future studies are recommended to further test the therapeutic potential of Src family inhibitors for the treatment of castration-resistant PCa.

## Supplementary information

Additional file 1. **Figure S1.** Prostate cancer cell-myofibroblast interaction and cytokine secretion in co-culture systems. (A) Proliferation of WPMY-1 treated with conditional media (CM) from prostate cancer cell line DU145 was measured with SRB. Viability of WPMY-1 control was normalized as 100%. (B) mRNA level of FAPa in WPMY-1 co-cultured with DU145 in transwell system was determined with qPCR, which of WPMY-1 untreated control was normalized as 1. (C) Proliferation of prostate cancer cell lines treated with conditional media (CM) from naïve WPMY-1 (CM-W) or WPMY-1 pre-activated by the conditioned medium of corresponding prostate cancer cells (i.e. CM-WL/CM-WP/CM-WD, the last letter indicates prostate cancer cell line). (D~E) Cytokines profiles in PCa-WPMY-1 co-cultured systems. The cytokines concentration was normalized with numbers of cells. W + L: WPMY-1 and LNCaP; 2D and 4D: 2 days and 4 days. D: CM-PC3 treated WPMY-1 vs. naïve WPMY-1 control; E: WPMY-1 and LNCaP transwell co-culture system vs. naïve WPMY-1 control. Data are represented as mean ± SD of triplicates from a representative triplicate experiment. Histograms shows quantitative results and *p* value was determined by ANOVA or t test, asterisks indicate *p < 0.05, **p < 0.01. **Figure S2.** Src family kinases signaling pathway activation is attenuated in CXCL1 treated prostate cancer cells using CXCR1/2 antagonist SCH527123. DU145 were treated with CXCL1 alone or supplemented with SCH527123 for indicated time, phosphorylation of Src, FAK and Paxillin were determined using western blotting. Experiments were repeated three times and representative blots were demonstrated. **Table S1.** CXCL1 associated genes in prostate cancer (Top ten). **Table S2.** Clinical and pathological characteristics of prostate cancer patients. **Table S3.** Correlation of CXCL1, CD177 and LCN2 expression in prostate cancer tissues. **Table S4.** Correlation of CXCL1 expression and clinical-pathological characteristics of prostate cancer patients. **Table S5.** CXCL1 is an independent prognosis factor of biochemical recurrence after radical prostatectomy. (DOC 1246 kb)

## Abbreviations

CM: Conditioned medium
CXCL1: Chemokine (C-X-C motif) ligand 1
hNEP: Human neutrophils
LCN2: Lipocalin-2
