# Supplementary material for: CXCL1-LCN2 paracrine axis promotes progression of prostate cancer via the Src activation and epithelial-mesenchymal transition
Source: Cell Commun Signal. 2019 Sep 10;17:118. doi: 10.1186/s12964-019-0434-3 (PMC6734451; doi:10.1186/s12964-019-0434-3)
Supplement: Supplementary file 1 — Figure S1. Prostate cancer cell-myofibroblast interaction and cytokine secretion in co-culture systems. (A) Proliferation of WPMY-1 treated with conditional media (CM) from prostate cancer cell line DU145 was measured with SRB. Viability of WPMY-1 control was normalized as 100%. (B) mRNA level of FAPa in WPMY-1 co-cultured with DU145 in transwell system was determined with qPCR, which of WPMY-1 untreated control was normalized as 1. (C) Proliferation of prostate cancer cell lines treated with conditional media (CM) from naïve WPMY-1 (CM-W) or WPMY-1 pre-activated by the conditioned medium of corresponding prostate cancer cells (i.e. CM-WL/CM-WP/CM-WD, the last letter indicates prostate cancer cell line). (D~E) Cytokines profiles in PCa-WPMY-1 co-cultured systems. The cytokines concentration was normalized with numbers of cells. W + L: WPMY-1 and LNCaP; 2D and 4D: 2 days and 4 days. D: CM-PC3 treated WPMY-1 vs. naïve WPMY-1 control; E: WPMY-1 and LNCaP transwell co-culture system vs. naïve WPMY-1 control. Data are represented as mean ± SD of triplicates from a representative triplicate experiment. Histograms shows quantitative results and p value was determined by ANOVA or t test, asterisks indicate *p < 0.05, **p < 0.01. Figure S2. Src family kinases signaling pathway activation is attenuated in CXCL1 treated prostate cancer cells using CXCR1/2 antagonist SCH527123. DU145 were treated with CXCL1 alone or supplemented with SCH527123 for indicated time, phosphorylation of Src, FAK and Paxillin were determined using western blotting. Experiments were repeated three times and representative blots were demonstrated. Table S1. CXCL1 associated genes in prostate cancer (Top ten). Table S2. Clinical and pathological characteristics of prostate cancer patients. Table S3. Correlation of CXCL1, CD177 and LCN2 expression in prostate cancer tissues. Table S4. Correlation of CXCL1 expression and clinical-pathological characteristics of prostate cancer patients. Table S5. CXCL1 is [file 12964_2019_434_MOESM1_ESM.doc]

**
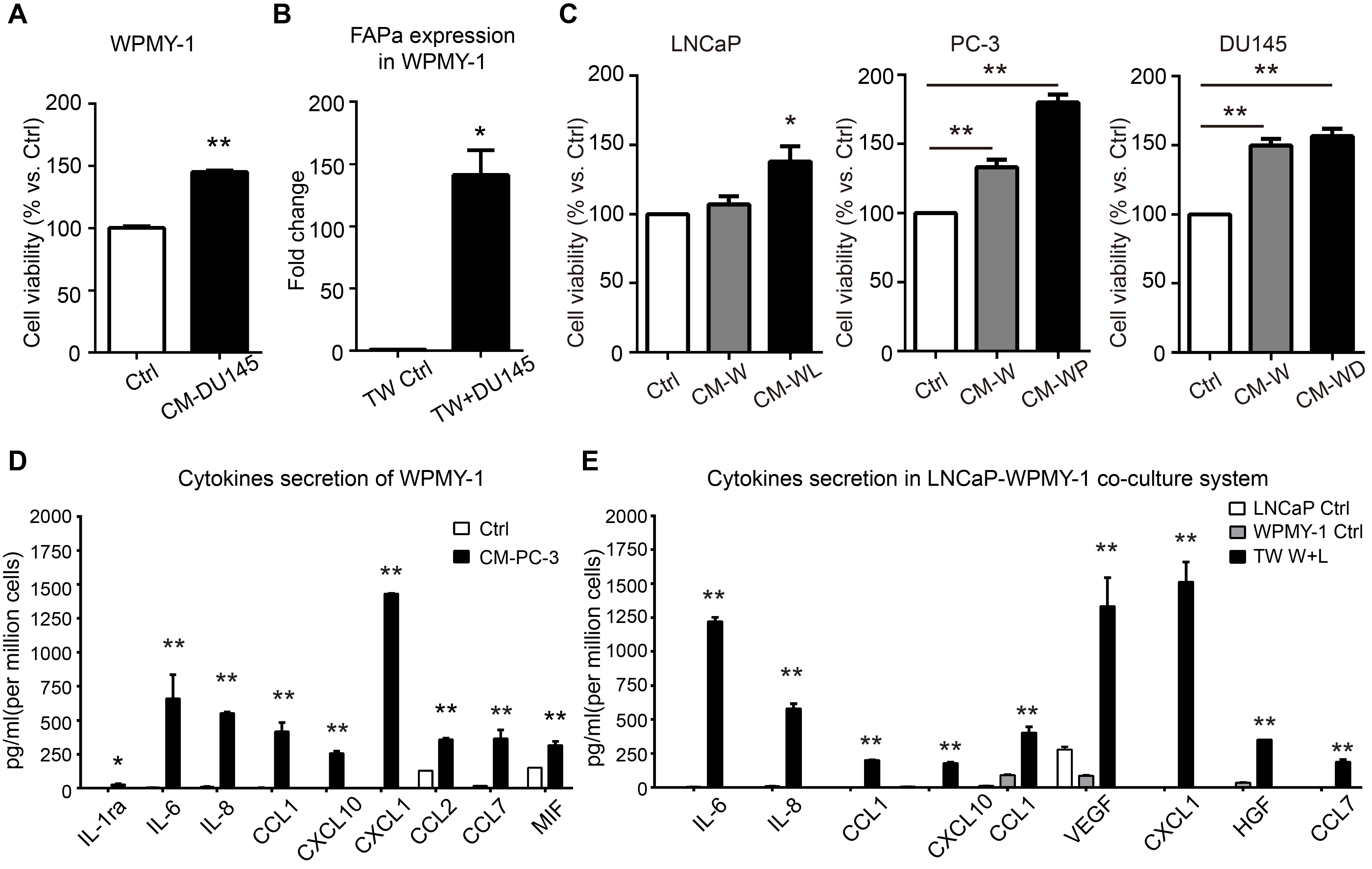
Figure S1** **Prostate cancer cell-myofibroblast interaction and cytokine secretion in co-culture systems.** (A) Proliferation of WPMY-1 treated with conditional media (CM) from prostate cancer cell line DU145 was measured with SRB. Viability of WPMY-1 control was normalized as 100%. (B) mRNA level of FAPa in WPMY-1 co-cultured with DU145 in transwell system was determined with qPCR, which of WPMY-1 untreated control was normalized as 1. (C) Proliferation of prostate cancer cell lines treated with conditional media (CM) from naïve WPMY-1 (CM-W) or WPMY-1 pre-activated by the conditioned medium of corresponding prostate cancer cells (i.e. CM-WL/CM-WP/CM-WD, the last letter indicates prostate cancer cell line). (D~E) Cytokines profiles in PCa-WPMY-1 co-cultured systems. The cytokines concentration was normalized with numbers of cells. W+L: WPMY-1 and LNCaP; 2D and 4D: two days and four days. D: CM-PC3 treated WPMY-1 vs. naïve WPMY-1 control; E: WPMY-1 and LNCaP transwell co-culture system vs. naïve WPMY-1 control. Data are represented as mean±SD of triplicates from a representative triplicate experiment. Histograms shows quantitative results and *p* value was determined by ANOVA or t test, asterisks indicate **p* < 0.05, ***p* < 0.01.


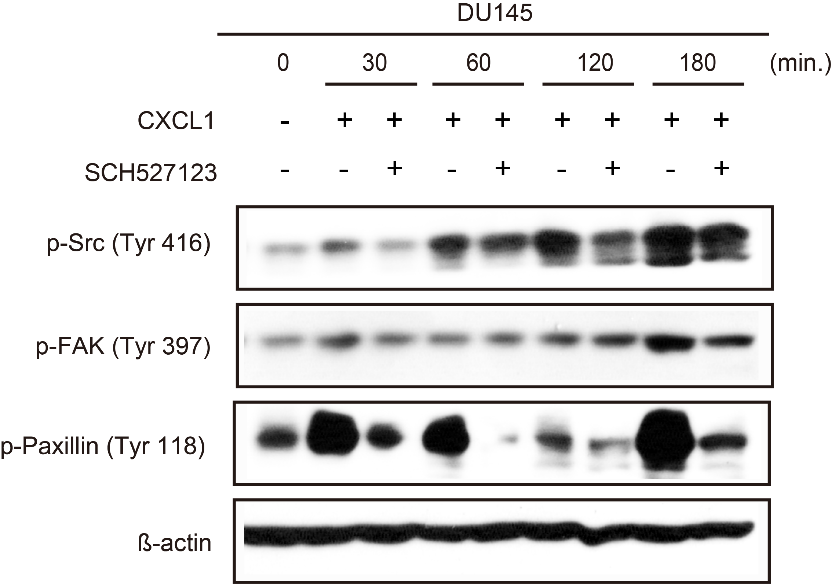


**Figure S2 Src family kinases signaling pathway activation is attenuated in CXCL1 treated prostate cancer cells using CXCR1/2 antagonist SCH527123.** DU145 were treated with CXCL1 alone or supplemented with SCH527123 for indicated time, phosphorylation of Src, FAK and Paxillin were determined using western blotting. Experiments were repeated three times and representative blots were demonstrated.

**Table S1. CXCL1 associated genes in prostate cancer (Top ten).**

| No. | Gene symbol | Gene title | Probe | P value | Protein type |
| --- | --- | --- | --- | --- | --- |
| 1 | CXCL6 | C-X-C motif ligand 6 | 206336_at | 3.69E-16 | chemokine |
| 2 | CXCL2 | C-X-C motif ligand 2 | 209774_x_at | 1.60E-14 | chemokine |
| 3 | IL8 | Interleukin 8 | 211506_s_at | 9.22E-11 | Cytokine/chemokine |
| 4 | PLAU | Urokinase-type plasminogen activator | 205479_s_at | 1.51E-09 | Serine protease |
| 5 | LCN2 | Lipocalin-2 | 212531_at | 5.29E-08 | Iron-trafficking protein |
| 6 | THBD | Thrombomodulin | 203887_s_at | 7.09E-08 | Cofactor for thrombin |
| 7 | OLFM4 | Olfactomedin 4 | 212768_s_at | 1.78E-07 | Anti-apoptotic factor/matrix glycoprotein |
| 8 | DKK1 | Dickkopf-related protein 1 | 204602_at | 2.63E-07 | WNT signaling pathway inhibitor |
| 9 | LGALS3 | Galectin-3 | 208949_s_at | 6.65E-07 | lectin family member |
| 10 | NTN4 | Netrin 4 | 223315_at | 9.18E-07 | Laminins related proteins |

**Table S2. Clinical and pathological characteristics of prostate cancer patients.**

| **Variables** | **Mean or Median ± SD (Range or %)** |
| --- | --- |
| Age at diagnosis, yr. | 66 (49~79) |
| Preoperative PSA, ng/ml | 26.2 (3.3~214.0) |
| Follow-up time, month | 69.1 (5~151) |
| No. of biochemical recurrence, n (%) | 48 (40.7) |
| Gleason score, n (%) |  |
| ≤6 | 30(25.4) |
| 3+4=7 | 25 (21.2) |
| 4+3=7 | 30 (25.4) |
| 8 | 21 (17.8) |
| 9-10 | 12 (10.2) |
| Adverse pathologic features, n (%) |  |
| Positive margins | 13 (11.0) |
| Seminal vesicle invasion | 22 (18.6) |
| Extracapsular extension | 14 (11.9) |
| Lymph node invasion | 4 (3.4) |
| CXCL1 expression score, n (%) |  |
| Negative | 23 (19.5) |
| Low | 9 (7.6) |
| Moderate | 43 (36.4) |
| High | 43 (36.4) |
| LCN2 expression score, n (%) |  |
| Negative | 51 (43.2) |
| Low | 11 (9.3) |
| Moderate | 34 (28.8) |
| High | 22 (18.6) |
| MPO expression score, n (%) |  |
| Negative | 84 (71.2) |
| Low | 22 (18.6) |
| Moderate | 11 (9.3) |
| High | 1 (0.8) |
| CD177 expression score, n (%) |  |
| Negative | 58 (49.2) |
| Low | 19 (16.1) |
| Moderate | 40 (33.9) |
| High | 1 (0.8) |

**Table S3. Correlation of CXCL1, CD177 and LCN2 expression in prostate cancer tissues.**

|  |  | CXCL1 | CD177 | LCN2 |
| --- | --- | --- | --- | --- |
| CXCL1 | Pearson correlation | 1 | 0.185 | 0.208 |
|  | Sig. (2-tailed) | - | 0.043 | 0.023 |
| CD177 | Pearson correlation | 0.185 | 1 | 0.376 |
|  | Sig. (2-tailed) | 0.043 | - | 0.000 |
| LCN2 | Pearson correlation | 0.208 | 0.376 | 1 |
|  | Sig. (2-tailed) | 0.023 | 0.000 | - |

**Table S4. Correlation of CXCL1 expression and clinical-pathological characteristics of prostate cancer patients.**

| Variables | CXCL1 expression (n) | | | | *P* value |
| --- | --- | --- | --- | --- | --- |
|  | Negative | Low | Mid | High |  |
| PSA (ng/ml) |  |  |  |  | 0.616 |
| <20.0 | 11 | 8 | 30 | 20 |  |
| ≥20.0 | 6 | 7 | 16 | 18 |  |
| APE |  |  |  |  | 0.507 |
| W/O APE | 14 | 14 | 33 | 28 |  |
| With APE | 3 | 2 | 14 | 11 |  |
| Gleason score |  |  |  |  | 0.399 |
| ≤6 | 3 | 5 | 16 | 9 |  |
| 7 | 11 | 8 | 21 | 15 |  |
| ≥8 | 17 | 15 | 47 | 39 |  |
| Stage |  |  |  |  | 0.369 |
| II | 15 | 14 | 33 | 26 |  |
| III | 1 | 1 | 8 | 8 |  |
| IV | 1 | 0 | 6 | 5 |  |
| BR |  |  |  |  | 0.001 |
| W/O BR | 12 | 11 | 35 | 13 |  |
| With BR | 5 | 4 | 12 | 26 |  |

**Table S5. CXCL1 is an independent prognosis factor of biochemical recurrence after radical prostatectomy.**

| Variables | Biochemical recurrence | | | Univariate analysis | | Multivariate analysis | |
| --- | --- | --- | --- | --- | --- | --- | --- |
|  | Groups | N | Median BFS (mo.) | P value | HR (95% CL) | P value | HR (95% CL) |
| Total |  | 47 | 72 |  |  |  |  |
| Preoperative PSA | <20 | 25 | Undefined | - | 1 |  |  |
|  | ≥20 | 21 | 72 | NS | 1.396 (0.779, 2.571) | NS | 1.581 (0.824, 3.032) |
| Gleason Score | ≤6 | 11 | 102 | - | 1 |  |  |
|  | 7 | 23 | 81 | NS | 1.346 (0.655, 2.767) | NS | 1.412 (0.632, 3.155) |
|  | ≥8 | 13 | 26 | NS | 1.276 (0.570, 2.858) | NS | 1.012 (0.359, 2.856) |
| APF | W/O | 33 | 102 | - | 1 |  |  |
|  | With | 14 | 72 | NS | 1.193 (0.635, 2.243) | 0.043 | 2.895 (1.035, 8.096) |
| Stage | II | 35 | 101.8 | - | 1 |  |  |
|  | III | 9 | 57.6 | NS | 2.313 (0.699, 7.658) | 0.013 | 7.555 (1.541, 37.045) |
|  | IV | 3 | Undefined | NS | 3.747 (0.986, 14.237) | 0.026 | 5.161 (1.222, 21.789) |
|  |  |  |  |  |  |  |  |
| CXCL1 expression | negative | 5 | 65 |  | 1 |  | 1 |
|  | low | 4 | 69 | NS | 0.912 (0.244, 3.405) | NS | 0.840 (0.205, 3.443) |
|  | medium | 12 | 67 | NS | 0.841 (0.296, 2.390) | NS | 1.035 (0.339, 3.155) |
|  | high | 26 | 26 | 0.012 | 3.418 (1.308, 8.932) | 0.010 | 4.133 (1.407, 12.139) |
| LCN2  expression | negative | 10 | Undefined | - | 1 |  |  |
|  | low | 4 | Undefined | NS | 0.597 (0.187, 1.909) | NS | 1.004 (0.279, 3.651) |
|  | medium | 18 | 102 | NS | 1.553 (0.716, 3.365) | NS | 1.135 (0.478, 2.695) |
|  | high | 15 | 56 | NS | 1.945 (0.872, 4.339) | NS | 2.124 (0.834, 5.410) |
